# Challenges and future directions in LC-MS-based multiclass method development for the quantification of food contaminants

**DOI:** 10.1007/s00216-020-03015-7

**Published:** 2020-11-13

**Authors:** David Steiner, Alexandra Malachová, Michael Sulyok, Rudolf Krska

**Affiliations:** 1 FFoQSI GmbH – Austrian Competence Centre for Feed and Food Quality, Safety and Innovation, Technopark 1C, 3430 Tulln, Austria; 2 Institute of Bioanalytics and Agro-Metabolomics, Department of Agrobiotechnology IFA-Tulln, University of Natural Resources and Life Sciences, Vienna (BOKU), Konrad-Lorenz-Str. 20, 3430 Tulln, Austria; 3 Institute for Global Food Security, School of Biological Sciences, Queens University Belfast, University Road, Belfast, Northern Ireland BT7 1NN UK

**Keywords:** Multiclass, Residues, Contaminants, Food safety, HRMS, MS/MS

## Abstract

Monitoring of food contaminants and residues has undergone a significant improvement in recent years and is now performed in an intensive manner. Achievements in the area of chromatography-mass spectrometry coupling techniques enabled the development of quantitative multi-target approaches covering several hundred analytes. Although the majority of methods are focusing on the analysis of one specific group of substances, such as pesticides, mycotoxins, or veterinary drugs, current trends are going towards the simultaneous determination of multiclass compounds from several families of contaminants and residues. This work provides an overview of relevant multiclass concepts based on LC-MS/MS and LC-HRMS instruments. Merits and shortcomings will be critically discussed based on current performance characteristics of the EU legislation system. In addition, the discussion of a recently developed multiclass approach covering >1000 substances is presented as a case study to illustrate the current developments in this area.

## Introduction

Sustaining a safe global food supply is a fundamental need and a dynamic process. Besides food-borne bacteria, parasites, and diet-related non-communicable diseases, food allergens, antibiotic resistance, endocrine-active pesticides, and mycotoxins including their derivatives are emerging threats to food safety [1]. The increasing food market globalization as integral part of the global protein supply chain and climate change are major challenges in monitoring and tracing food contaminants from farm-to-fork [2]. Thus, environmental analysis is facing the problem to control and assess the risks related to mixtures of emerging contaminants, which are constantly changing [3]. A recent review on the chronic health risk for the European consumer has revealed that the co-exposure to a mixture of potentially genotoxic-carcinogenic substances, such as food process contaminants, at potential high-risk levels is alarming. It can be expected that the combined risk from the co-exposure of a mixture of contaminants with a similar mode of action is significantly higher compared to risks assessed for single chemicals [4]. Combined exposure assessment, which is not yet executed by the European Food Safety Authority (EFSA) for chemical mixtures, but for chemicals with an existing group TDI (tolerable daily intake), is typically following a comparison of occurrence data on chemicals with human consumption data and using concentration data for the respective ecological area. A bottleneck of this approach is related to differing quality and quantity of the data for different compounds, which hampers routine risk assessment of chemical mixtures based on occurrence data obtained from different analytical methods [5]. In order to conduct a reliable risk assessment of co-exposures to potentially harmful substances, novel developments in mixture toxicity data and quantitative target analysis are required [4]. Hence, there is a major trend in analytical chemistry towards the development of precise and reliable but also considerably faster and cheaper methods for the trace analysis of multiple target and non-target organic compounds in complex food and feed [2, 6]. Among the most investigated organic contaminants and residues are natural toxins (e.g., mycotoxins and plant toxins) in nuts and cereals [7, 8], pesticides in fruits and vegetables [9, 10], and veterinary drugs in meat and animal products [11, 12]. However, the number of analytical approaches combining several classes of contaminants within one analytical run is still comparatively scarce. The majority of multi-target publications are either focusing on one single substance class, or the substance class is segmented into subcategories, e.g., sulfonamides, tetracyclines, or penicillins (in case of veterinary drugs) in order to obtain a multiclass scope. Therefore, the focus of this article is twofold (i) to provide an overview of existing analytical multi-compound approaches including new trends in multiclass method development and (ii) to illustrate limitations and challenges in their broad applicability.

## The legislation system of the European Union

In order to minimize contaminated foodstuff, the European Union (EU) has taken measures to control the amounts of environmental contaminants which may hamper the quality of food and imply a risk to the European community [6]. The rationale behind an effective assurance of food safety is based on an integrated “farm-to-fork” approach, applicable for both microbial food contamination and to potentially harmful residues and contaminants [13]. As a legislative consequence, the latter were regulated with several maximum limits (MLs) and maximum residue limits (MRLs) for numerous substances in a large number of matrices [2]. Table 1 provides an overview of the existing legislative framework for the regulation of food contaminants adopted by the European Commission (EC). Within the Commission Regulations (EC) 1881/2006, 396/2005, and 37/2010, and their current amendments, MRLs for mycotoxins, MLs for pesticides, and veterinary drugs in different food commodities are set [14, 16, 17].

**Table 1:** European legislative framework for the regulation of food contaminants

Food contaminants	MRLs/MLs	Matrices	EC regulations
Mycotoxins	0.025–2000 μg/kg	Foodstuff and animal feed	Reg. 1881/2006, Reg. 32/2002 [14, 15]
Pesticides	10 μg/kg (default value)	Food and feed of plant and animal origin	Reg. 396/2005 [16]
Veterinary drugs	0.05–20,000 μg/kg	Foodstuffs of animal origin	Reg. 37/2010 [17]

In addition, EC Directive 32/2002 implies maximum levels for undesirable substances like dioxins, or aflatoxin B_1_ in animal feed [15]. Since these regulatory limits define the permitted amount of chemical pollutants in food and feed, they represent the indicators for the analytical capabilities in terms of method performance for different analyte/matrix combinations. These performance requirements have to be achieved by confirmatory methods, which are approaches enabling an unequivocal identification of substances and their quantification at the level of interest [18]. However, these requirements pose a dual challenge with respect to partially extreme low limits of quantification (LOQ), e.g., 0.025 μg/kg for aflatoxin M_1_ in infant formulae and additionally a broad working range, e.g., in case of mycotoxins and veterinary drugs, which requires high instrumental performance in both ultra-low and high concentration levels.

## Multi-analyte approaches: an overview

In recent years, the coupling of chromatography-mass spectrometry techniques has become state-of-the-art for ultra-trace analysis due to significant achievements in terms of sensitivity [3]. This has resulted in a considerable progress in food analysis which allowed the simultaneous monitoring of compliance with the legally permitted maximum values within a significantly reduced analytical turnaround time [6]. A combination of gas chromatography (GC) or liquid chromatography (LC) with tandem mass spectrometry (MS/MS) or high-resolution mass analyzer (HRMS) such as time of flight (TOF) or Orbitrap is the most common coupling technique in routine analysis. However, a comprehensive comparison between GC-MS and LC-MS for 500 high priority pesticides, which was conducted by Alder et al., has revealed an advantage for LC-based techniques in terms of wider scope, better selectivity, and increased sensitivity. In addition, most determinations are able to be performed without derivatization with LC-MS/MS, manifesting it as the preferred combination for residue analysis [19]. The extended use of this instrumentation led to numerous multi-compound methods in the area of mycotoxin, pesticide, and veterinary drug analysis [7–12]. However, the first comprehensive method combining several substance classes within one analytical procedure was designed by Mol et al. in the year 2008 [20]. Since then, the number of so-called multiclass methods has increased considerably, as a comprehensive overview by Turnipseed and Jayasuriya has recently revealed [21]. Selected LC-MS-based multiclass applications for food and feed are provided in Table 2. For mass analyzers, there is a clear trend towards MS/MS and Orbitrap in combination with an electrospray ionization (ESI) interface. The main difference between these two mass analyzers is based on the monitoring algorithm which is usually performed in multiple reaction monitoring (MRM) mode within MS/MS and in full-scan mode within Orbitrap or other HRMS instruments. In recent years, HRMS devices were reported to provide poorer sensitivity compared to MS/MS which resulted in the use of these instrumentations as screening devices for untargeted applications. This weakness was reduced by several technological improvements like higher resolution power for the reduction of isobaric interferences with matrix components, the introduction of new ion transition devices, and advances in detection technology [32].

**Table 2:** LC-MS applications for multiclass determination in food and feed analysis

Compounds	Matrix	Sample preparation	Stationary phase	Detection	Run time	LOQ (μg/kg)	Reference
Mycotoxins, pesticides, veterinary drugs, plant toxins, bacterial metabolites (1467)	Compound feed (cattle, chicken)	Dilute and shoot acetonitrile/water/formic acid 79:20:1, v/v/v	Gemini C_18_ (150 mm × 4.6 mm, 5 μm)	MS/MS (ESI +/−)	21 min (two runs)	0.1–900	[22]
Pesticides, veterinary drugs, metabolites, environmental contaminants (262)	Beef (bovine muscle)	QuEChERS	HSS T3 C_18_ (100 mm × 2.1 mm × 1.8 μm)	MS/MS (ESI +/−)	10.5 min	10–500	[23]
Mycotoxins, bacterial metabolites, plant toxins (> 500)	Wheat, maize, figs, dried grapes, walnuts, pistachios, almonds	Dilute and shoot acetonitrile/water/acetic acid 79:20:1, v/v/v	Gemini C_18_ (150 mm × 4.6 mm, 5 μm)	MS/MS (ESI +/−)	21 min (two runs)	0.02–1900	[24]
Pesticides, veterinary drugs, food packaging contaminants, mycotoxins, perfluorinated compounds, nitrosamines, sweeteners (633)	Baby food, orange, tomato	QuEChERS	Zorbax RRHD Eclipse-Plus C_18_ (50 mm × 2.1 mm × 1.8 μm)	QTOF (+/−)	15 min (two runs)	0.06–420	[25]
Pesticides, mycotoxins, plant toxins (389)	Leek, wheat, tea	QuEChERS	Accucore™ aQ C_18_ (150 mm × 2.1 mm, 2.6 μm)	Exactive-Orbitrap (ESI +/−)	10.5 min (two runs)	0.2–5000	[26]
Veterinary drugs, mycotoxins, pesticides, hormones (226)	Bovine and porcine muscle	SLE acetonitrile/ethanol 5:1, v/v, purification by low temperature and d-SPE	Acquity HSS-T_3_ (100 mm × 2.1 mm, 1.8 μm)	MS/MS (ESI +/−)	12 min (two runs)	0.05–10	[27]
Pesticides, veterinary drugs (>350)	Honey	SLE acetonitrile/1% formic acid	Hypersil Gold aQ C_18_ (100 mm × 2.1 mm, 1.9 μm)	Exactive-Orbitrap (ESI +)	14 min	10–250	[28]
Mycotoxins and pesticides (69)	Wine	SPE (Oasis HLB) elution with methanol	Zorbax Rapid Resolution Eclipse XDB-C_18_ (50 mm x 4.6 mm, 1.8 μm)	TOF (+)	18 min	0.1–13	[29]
Pesticides, plant alkaloids, veterinary drugs (118)	Corn silage, muscle and liver tissue, whole milk	QuEChERS	Hypersil Gold aQ C_18_ (50 mm × 2.1 mm, 1.9 μm)	Exactive-Orbitrap (ESI +)	12 min	1–5000	[30]
Mycotoxins, pesticides, and biopesticides (90)	Cucumber, wheat, red wine	QuEChERS	Acquity BEH C_18_ (100 mm × 2.1 mm, 1.7 μm)	MS/MS (ESI +)	13 min	3–10	[31]
Pesticides, mycotoxins, plant toxins, veterinary drugs (258)	Compound feed, honey	Dilute and shoot water/acetonitrile/1% formic acid 5:15, v/v	Acquity BEH C_18_ (100 mm × 2.1 mm, 1.7 μm)	MS/MS (ESI +/−)	19.5 min	10–250	[20]

*LOQ*, limit of quantification; *QuEChERS*, quick, easy, cheap, efficient, rugged, safe; *SLE*, solid-liquid extraction; *SPE*, solid-phase extraction; *MS/MS*, tandem mass spectrometry; *ESI*, electrospray ionization; *(Q)TOF*, (quadrupole)-time-of-flight

**Fig. 1:**
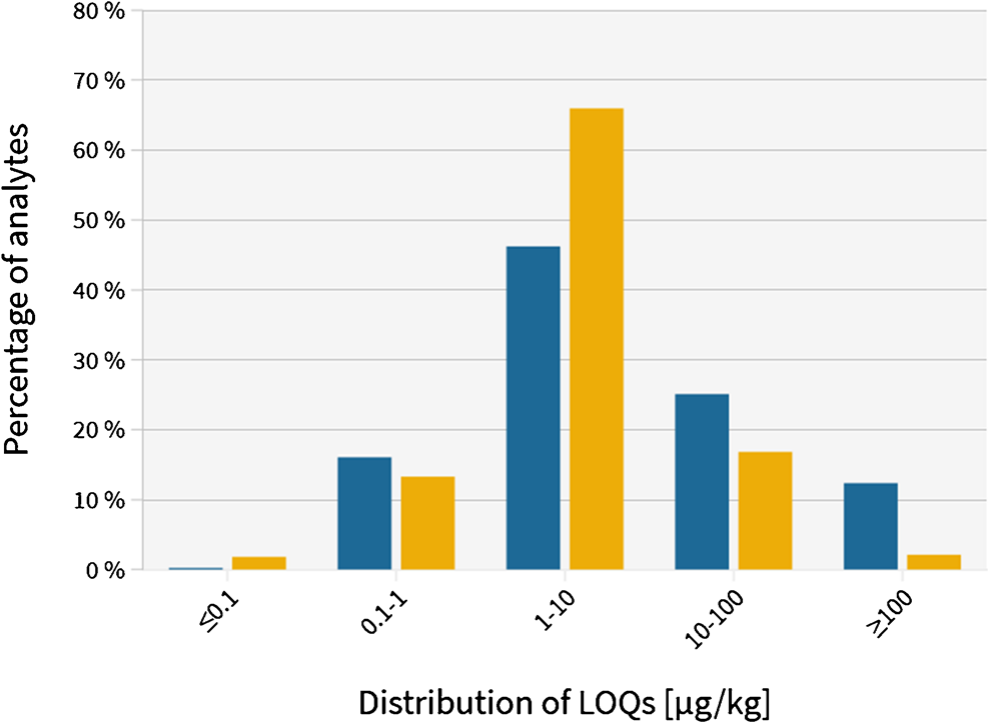
Performance comparison between HRMS (blue) and MS/MS (orange) instruments based on > 3800 LOQ results for pesticides, mycotoxins, veterinary drugs, and plant toxins [22, 24, 26, 30]. The y-axis represents the percentage of analytes, and the x-axis represents the distribution of LOQs in μg/kg

As a result, the competitiveness of HRMS has significantly increased which is demonstrated by a performance comparison between HRMS and MS/MS in Fig. 1. Within the scope of investigation, over 3800 LOQ results (following a EURACHEM based calculation) for mycotoxins, pesticides, and veterinary drugs covered in one method were compared and did not reveal a significant difference, leading to the conclusion that HRMS strongly competes with classical MS/MS concepts and is able to handle routine trace level analysis. In addition, with HRMS concepts, data can be reprocessed a posteriori which means a retrospective evaluation of compounds based on their isotopic profile and accurate mass. However, this retrospective investigation is associated with very large data volumes, which may hamper the routine applicability of this approach as data storage and traceability pose a major limitation [33]. In fact, the majority of quantitative targeted techniques are still based on MS/MS instruments as it is applicable for ultra-trace analysis and allows a monitoring of > 1000 compounds in one analytical run [22].

## Challenges and limitations

### Data acquisition in MS/MS

The realization of such comprehensive approach requires adjustments to the acquisition algorithm, since MRM-based targeted data acquisition is limited to the number of analytes (~ 200 compounds) that can be detected within one run [20, 32]. The limiting factor is based on the number of contemporary transitions as well as on the lowest possible dwell times (t_Dwell_), as the number of data points per peak and the acquisition or cycle time (t_Cycle_) is defined by this parameter. In order to increase the number of detectable compounds (< 500 analytes) and to ensure an appropriate amount of t_Dwell_ (≥ 10 msec) and data points per peak (10–15), data acquisition has to be performed following a scheduled reaction monitoring (sMRM) algorithm. Within sMRM, each analyte is measured within a predefined time window (t_Window_) reflecting the expected retention time [34]. The reduction of t_Window_ and thus a decrease of t_Cycle_ lead to a significant improvement of t_Dwell_ as it follows an automated calculation by dividing t_Cycle_ through the number of concurrent MS/MS transitions. This enables a significant expansion of the method’s scope. Since retention times commonly show neither a relative nor a consistent absolute stability especially for acid and alkaline compounds, t_Window_ must be set up thoroughly and readjustments after long sample series has to be taken into account [32]. In addition, frequent changes of eluents and acquisition methods in the LC-MS/MS system should therefore be avoided.

### Sample preparation

For the determination of multiple chemical residues and contaminants in food and feed, the major bottleneck in any analytical procedure within the laboratory remains as sample preparation [30]. In the past few years, the trend in multi-compound analysis moved towards generic extraction protocols which are applicable for a broad range of food and feed commodities including complex matrices [35]. Their main characteristic is based on a high sample throughput enabled by using small sample amounts and reduce the volume of organic solvents. One of the most popular and frequently used extraction techniques is QuEChERS (quick, easy, cheap, effective, rugged, and safe) which was developed by Anastassiades et al. in 2003 [36]. Originally developed for the extraction of organic compounds such as pesticides, QuEChERS has become one of the most prominent multiclass extraction techniques, since it is applicable to a large number of different substance classes [26, 30, 31]. However, a comprehensive comparison of seven different generic extraction protocols including QuEChERS, which was conducted by Mol et al., has revealed an even more straightforward “dilute and shoot”-based approach as most suitable for a variety of matrices. The default method of choice was based on water/acetonitrile/1% formic acid [20]. However, it is obvious that a simplification of the extraction protocol poses some challenge considering the achievement of appropriate extraction efficiencies for all analyte/matrix combinations. Especially very polar compounds might be lost following a QuEChERS protocol, and extraction yields based on dilute and shoot protocols might be insufficient for some compounds as extraction solvents do not cover all analyte-specific requirements such as pH optima.

### Matrix effects

Although high extraction yields are a top priority in method development, co-extraction of matrix inherent components such as carbohydrates, proteins, and fat may negatively contribute to the accurate quantitative analysis, reduce the lifetime of the analytical column, and pollute the entrance to the mass spectrometer. These sample-dependent effects cause a suppression or enhancement (SSE) of the analyte response within the ionization process [20]. In order to reduce or compensate these effects, classical QuEChERS protocols are combined with a subsequent sample clean-up step by using, e.g., d-SPE, PSA, or a C_18_ bulk sorbent [11, 12, 26]. However, the broad applicability of these unspecific clean-up procedures is limited, since they are not compatible to all compounds and some target analytes might be lost during this step (e.g., fumonisins after PSA clean-up) [22]. Also, an increase in dilution factors might not be suitable especially to current HRMS instrumentations as the analytical result could be hampered due to a lack of sensitivity [35]. In routine analysis, the so-called stable isotope dilution assays (SIDA) are very common. These approaches are based on the use of small amounts of isotopically labeled internal standards (ISTD) which are simultaneously injected with sample extracts in the autosampler. Despite the powerful compensation of matrix effects using SIDA, its wide application is limited since only a small number of ISTD is commercially available [37]. Furthermore, the procedure matched (applying the internal standard to the raw material prior extraction) use for ISTDs also pose an economic challenge, since certified internal standard solutions are at higher price level and thus not affordable for many research groups. Another frequently used approach is the preparation of the so-called matrix-matched calibration (MMC) standards as conducted by Dzuman et al. [26] SSEs can be effectively compensated by MMC, but the applicability for multiclass methods covering several hundred compounds is limited due to the lack of matrix reference materials which are entirely blank for all target analytes [24]. In addition, with increasing sample complexity which, e.g., applies for compound feed, the high intra-matrix variations cannot be compensated by using a “default” sample extract for MMC preparation [38]. Hence, the lack of matrix effect compensation and reduction strategies is the main limitation of an LC-MS-based multiclass approach as these effects cannot be removed efficiently.

### Feasibility of multiclass methods

As highlighted in Table 2, the majority of “modern” multiclass methods are either based on low-resolution MS/MS instruments, or high-resolution devices including Orbitraps and time-of-flight mass analyzers. With respect to the latter, one major advantage of HRMS is based on their easy adaption for non-targeted analysis, which opens possibilities for unexpected findings. In addition, retrospective analysis enables the identification of additional metabolites and transformation products which can provide distinct information on the influence of changing climate conditions to the occurrence of natural contaminants, such as mycotoxins [21]. However, traditional quantitative approaches based on HRMS are difficult to compare, but each mass analyzer (Orbitrap and TOF) has its specific merits. The major advantage of Orbitraps is based on the high mass resolving power (> 100,000 full width at half maximum) enabling a clear differentiation between target compounds and matrix interferents. Compared to TOF instruments, the main drawback of Orbitraps is based on the inverse relationship to resolution and lower scanning speed. Thus, a combination of TOF devices with highly efficient separation techniques (ultra-high performance liquid chromatography) could provide shorter run times [39]. Although different instrumentations (including low- and high-resolution devices) show distinct advantages and disadvantages with respect to multiclass method development, a simplified sample preparation protocol either based on QuEChERS [23, 25, 26, 30, 31], or dilute and shoot [20, 22, 24] will cause more similarity within the methods applied in different laboratories. This will allow a better comparison of data obtained from different analytical procedures, even for results near the limit of quantification. Compared to existing MLs for mycotoxins and MRLs for pesticides, and veterinary drugs, the sensitivity of current multiclass methods (based on different MS technologies) is sufficient to ensure LOQs which comply with the maximum permitted levels of contaminants and residues.

As exemplary illustrated in Table 3, multiclass approaches, either based on MS/MS [24] or Orbitrap [26] technology, meet existing method performance criteria and provide sufficient sensitivity to cover existing maximum limits for mycotoxins in unprocessed cereals, such as wheat. In contrast, matrices such as baby food are not yet feasible to be implemented within the scope of current multiclass concepts [25] without making compromises, as these sample types require ultra-sensitivity in order to comply with extreme low maximum levels. However, in terms of costs and manual workload, there is a potential reduction for multiclass methods compared to single analytical approaches which require specific sample preparation techniques and instrumentation.

**Table 3:** Performance of multiclass methods based on EC 401/2006

	Unprocessed cereals									
	EC 401/2006 [40]			EC 1881/2006 [14]	MS/MS [24]			Orbitrap [26]		
Analyte	μg/kg	rec %	RSD_r_ %	ML μg/kg	rec %	RSD_r_ %	LOQ μg/kg	rec %	RSD_r_ %	LOQ μg/kg
Aflatoxin B_1_	1–10	70–110	Horwitz	2	84	6	0.7	82	3	0.5
Aflatoxin B_2_	1–10	70–110	Horwitz	2	86	5	0.2	86	8	2.5
Aflatoxin G_1_	1–10	70–110	Horwitz	2	80	3	0.5	84	6	0.5
Aflatoxin G_2_	1–10	70–110	Horwitz	2	81	4	1.7	84	8	0.5
Deoxynivalenol	> 500	70–120	≤ 20	1750	102	13	n.a.	87	2	500
HT-2 toxin	>200	60–130	≤ 30	100*	100	7	5	80	7	12.5
Ochratoxin A	1–10	70–110	≤ 20	3	97	5	1.5	82	4	5
T-2 toxin	> 250	60–130	≤ 30	100*	98	4	2.4	89	6	0.5
Zearalenone	> 50	70–120	≤ 25	100	100	3	0.6	83	6	2.5

*Indicative level

*rec*, apparent recovery; *RSD*_*r*_, relative standard deviation (under repeatability conditions); *n.a.*, not available

## Multiclass goes beyond 1000 analytes

Recent efforts in the area of multiclass analysis have been concentrating both reducing time required for the analysis and increasing the number of analytes. Existing multiclass approaches [20, 26, 28] are rather limited with respect to the scope of target compounds which contain no more than 400 analytes. Very recently, our group developed an LC-MS/MS-based multiclass approach for the accurate quantification of > 1000 compounds from five major substance classes which was fully in-house validated for two compound feed matrices [22]. In order to minimize the overall analytical workload and ensure maximum compatibility to the broad scope of analytes, we chose a time- and cost-efficient “dilute and shoot” approach for sample preparation based on acetonitrile/water/1% formic acid. In an unprecedented way, we have optimized HPLC/UHPLC and MS/MS conditions with special focus on t_Dwell_ and t_Cycle_.

### Optimization of the (U) HPLC system

**Fig. 2:**
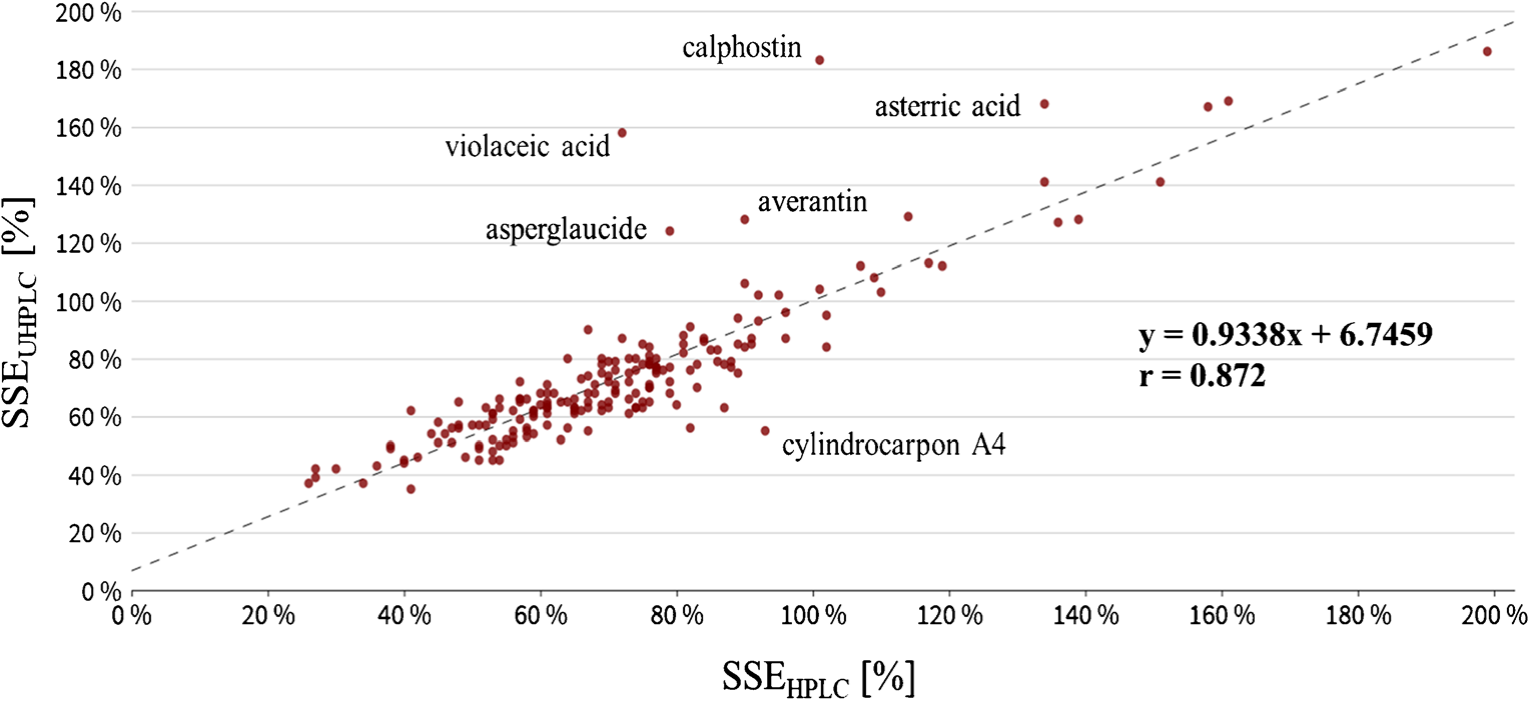
Comparison of matrix effects (SSE) under HPLC (x-axis) and UHPLC (y-axis) conditions. Results are based on five spiked cattle feed extracts with a set of 200 representative analytes. Statistical analysis revealed no significant difference neither for absolute (P_(T < =t)_ = 0.22) nor for relative (P_(F < =f)_ = 0.42) matrix effects

With respect to the chromatographic system applied within routine analysis, a clear trend is towards ultra-high-performance liquid chromatography (UHPLC) which is also evident to a large number of multiclass approaches [20, 26–31]. The advantage is characterized by columns of smaller diameters and particle size resulting in improved efficiency and reduced analysis time, as illustrated, e.g., between Dzuman et al. (10.5 min under UHPLC) and Sulyok et al. (21 min under HPLC) [24, 26, 41]. However, in order to achieve maximum performance for methods with this extent, two individual injections (one in positive and one in negative ionization mode) of the same sample are necessary [26]. In addition to the accelerated analytical turnaround time, UHPLC columns should provide benefits with respect to matrix effects through improved separation and reduction of overlapping events with co-eluting matrix components [42]. Contrary to these expectations, a comparison between spiked cattle feed extracts with a multi-compound standard for a selected set of representative analytes (200 compounds) revealed no clear advantage of UHPLC with respect to matrix effects, as these effects remained the same for the majority of analytes (Fig. 2).

**Fig. 3:**
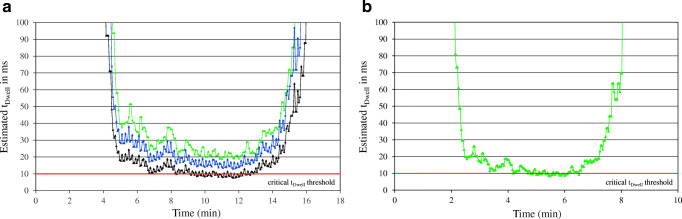
Estimated t_Dwell_ under HPLC (**a**) and UHPLC (**b**) conditions. Configurations for HPLC acquisition parameters consist of t_Window_ of 40, 40, and 30 s and t_Cycle_ of 1.0, 1.5, and 1.5 s for setup 1 (black), setup 2 (blue), and setup 3 (green) with a total run time of 21 min. UHPLC run time was adjusted to 10.5 min and the configuration consist of 0.8 s t_Cycle_, 15 s t_Window_ matching HPLC setup 3 (green)

Although peak resolution improved significantly, the benefits of UHPLC might be lost with increasing number of analytes (current UHPLC multiclass approaches cover a maximum of 400 compounds) as the improved peak resolution cannot prevent increasing overlapping events between hundreds of target analytes and co-extracted matrix components. In addition, UHPLC evokes the problem on achieving sufficient data points per peak and t_Dwell_, as narrowing the peak shapes requires significantly reduced t_Cycle_ in order to obtain a sufficient number of data points across the (narrower) chromatographic peak (Fig. 3b).

### Improvements of data acquisition setup

In order to minimize the overall measurement error by increasing t_Dwell_, precise adjustments to t_Cycle_ and t_Window_ were conducted and are highlighted in Fig. 3. Under HPLC conditions, an increase of t_Cycle_ to 1.5 s in combination with a t_Window_ of 30 s significantly (~ factor 2) improved t_Dwell_, since the number of concurrent MRM transitions in the most critical chromatographic time window was reduced.

Although these adjustments caused a sacrifice in terms of data points per peak (from 15 to 10 per 15-s peak width), significant improvements to the method precision (tested with a multi-compound standard near the instrumental LOQ under repeatability conditions) were observed. In contrast, matching of UHPLC acquisition parameters to the optimal conditions of HPLC did not lead to appropriate amounts of t_Dwell_, as a number of compounds still undercut the critical t_Dwell_ threshold of 10 ms. Based on the assumption that benefits of UHPLC with respect to matrix effects are limited with increasing number of target analytes and the overall measurement error might increase due to a lack of sufficient t_Dwell_, the final decision was made for HPLC as it is able to tackle scopes exceeding the previous amount of target analytes.

## Outlook

In recent years, the number of multiclass approaches covering up to 1000 compounds have steadily increased. Most applications follow a generic extraction protocol like QuEChERS or dilute and shoot in combination with LC-MS/MS or HRMS instruments. High sample throughput can be achieved by UHPLC-based systems as they ensure a significant reduction in analysis time. However, the broad applicability of such systems might be limited with the increasing number of target compounds, since a sufficient achievement of data points per peak and dwell times is not feasible. HPLC concepts may circumvent this issue as they provide enough t_Dwell_ despite a large number of target analytes, but this requires an appropriate and time-consuming adjustment of acquisition parameters.

It can be expected that the number of HRMS instrumentations applied in routine analysis will strongly increase in the next decade. However, in order to fully compete with MS/MS instruments, two significant adjustments are necessary: (i) expand the linear working range and (ii) make the devices affordable for a wide range of applications. Since matrix effects remain the major bottleneck in multiclass method development and compensation and reduction strategies are rather limited, only strong dilutions of crude sample extracts can lead to a significant reduction of these unwanted effects. In contrast to HRMS, MS/MS devices are able to tackle high dilution factors and have become state-of-the-art instruments for ultra-trace analysis due to strong improvements in terms of sensitivity. Additionally, HRMS instruments are often limited in their linear dynamic range compared to MS/MS which hampers the applicability of these instruments with respect to the broad working range applied in routine analysis. To represent a long-term alternative to MS/MS, it is necessary that HRMS instruments do not lose the connection in terms of sensitivity and expand their linear working range. With respect to the instrumentation and technology used, there is a price to be paid. The acquisition of new mass analyzers is accompanied by high financial expenses in addition to infrastructural costs in order to house and operate the system, or to ensure proper trainings for laboratory specialists. The broad use of MS/MS instruments has led to a dominance of these devices in routine analysis which in turn improved the affordability. Although the application of HRMS devices has also increased within the past years, we think they are still at a considerably higher price level. In addition, according to the experience of different working groups in our institute using both HRMS and MS/MS, the routine use of HRMS instruments requires a higher level of expertise compared to MS/MS as troubleshooting is more complicated and device issues occur more frequently. These drawbacks prevail the option of retrospective data analysis and a potential infinite number of analytes to be determined in full-scan mode. Hence, it is unlikely that HRMS instruments will pose a “paradigm shift” in the area of routine residue analysis as there is still a big margin of improvement. However, due to the major advantage of HRMS instruments of interrogating historical data of occurrence patterns, these devices will play an important role for measuring impacts of climate change.

In order to expand the scope of analytes monitored within the global food supply chain, validation guidelines and regulatory programs have to take the trends in multiclass method development into account and adapt the existing framework. It has been shown that method development and validation of > 1000 target compounds in complex matrices is feasible [22] from a technical perspective, but is associated with an overwhelming data management. To reduce the overall workload after an initial method validation, future performance guidelines have to strike a compromise in terms of method transferability to other matrices, and manual inspection of chromatograms during LOQ evaluation. A matrix independent LOQ estimation as suggested by Sulyok et al. [24] might be a suitable solution in order to keep the overall effort reasonable. In conclusion, it will be important that the regulatory framework opens new possibilities for comprehensive data analysis in order to enable a simultaneous monitoring of hundreds of contaminants and residues in as many food and feed commodities as possible.
